# Consistency‐Based Approach to Adjust for Multiplicity in Confirmatory Subgroup Analysis

**DOI:** 10.1002/sim.70154

**Published:** 2025-06-11

**Authors:** Qiqi Deng, Qian Li, Naitee Ting, Feng Yu

**Affiliations:** ^1^ Biostatistics Moderna, Inc Cambridge MA USA; ^2^ Biostatistics StatsVita, LLC Bethesda MD USA; ^3^ School of Mathematics University of Bristol Bristol UK

**Keywords:** biomarker, enrichment, multiplicity adjustment, subgroup, type I error inflation

## Abstract

With the advance of medical sciences and better understanding of human biological systems, the next generation of treatment has shifted toward personalized medicine. It is expected that personalized medicine, such as molecularly targeted anti‐cancer agents, is more efficacious in marker‐positive patients, while marker‐negative patients may or may not benefit from the treatment. Due to technology limitations in marker identification and incomplete understanding of the role of biomarkers in treatment effect, it is possible that the marker is not predictive. Therefore, it is often of interest to test the treatment on the overall population as well as the biomarker‐positive subgroup. Testing both the overall population and the biomarker‐positive subgroup introduces a multiplicity issue and leads to type I error inflation if not adjusted appropriately. The available multiplicity adjustment procedures may not consider the logic needed in the two tests. A new method is proposed by applying the logical connections between the two hypothesis tests, and arbitrages between different rejection regions to make the testing strategy not only powerful but also sensible.

## 1 Introduction

Despite the well‐defined intention‐to‐treat (ITT) patient population in clinical studies for a proposed indication, individual patients may not respond to the treatment homogeneously. A certain subgroup, for example, patients with a certain biomarker, may have a different response to a treatment from the general population. Beckman et al. (2011) [1] gave the example of Gefitinib, a treatment that initially failed in the full population but was later shown to have a strong beneficial effect in patients with certain mutations. This is one of the reasons why, in clinical trial protocols, subgroup analyses need to be pre‐planned. The subgroups can be defined by disease stages, certain demographic characteristics, or genetic information known as biomarkers. Many drug approvals by the FDA have only been granted to subgroups of the ITT population. In case the treatment effect has been demonstrated to benefit the overall population, and a certain subgroup's effect is larger than the effect in the overall ITT population, this subgroup's effect may be reflected in the drug label. Examples can be found in a summary report by Amatya et al. (2021) [2]. Both FDA and EMA have issued guidance on this topic [3, 4]. In 2019, the FDA published the most recent version of the guidance for the industry: Enrichment Strategies for Clinical Trials to Support Determination of Effectiveness of Human Drugs and Biological Products. This guidance specifically discusses strategies that can be considered to enrich certain subgroups in a study to enhance the efficiency of the study. It further considers certain subgroups that may potentially have better treatment effects. In the 2019 guidance to the industry on adaptive design [5], the FDA document discusses the adaptive enrichment strategy, which allows the trial to change the study population to a subgroup based on interim analyses. Suppose the development strategy is to register for both marker‐positive patients and overall patients, it helps to focus the recruitment of marker‐positive patients while adding some marker‐negative patients. FDA guidance on enrichment [3] indicates “FDA encourages the inclusion of some predictive marker‐negative patients in most trials intended to provide primary effectiveness support, unless earlier studies have established that the marker‐negative patients do not respond or a strong mechanistic rationale makes it clear that they will not respond.”

A biomarker is usually identified based on a drug's mechanism of action. Typical baseline biomarkers can be classified into “prognostic marker” and “predictive marker.” A prognostic marker is a biomarker to help identify patients who have better prospects of clinical outcomes, regardless of which specific intervention is being applied. In other words, patients with a positive value of this biomarker tend to have a better prognosis, regardless of the treatment they receive. For example, if it is generally believed that a younger patient would have a better prognosis than an older patient, as anticipated from the usual course of a certain disease, then age may be considered a prognostic biomarker, with “young age” as a positive marker value, and “old age” as negative. On the other hand, a “predictive biomarker” is treatment‐specific. That means, if a patient has a positive value on a predictive biomarker, then this patient is predicted to respond better than a patient with a negative value of this marker. For example, if a new drug is developed to target a particular genetic marker in the human body. It is known that most people with this marker tend to follow a particular disease process. By modulating such a disease process, this test drug helps improve patient response. Then, patients with this particular gene (marker‐positive) are predicted to have a better chance of disease improvement. A baseline biomarker can be prognostic, predictive, neither, or both. For such a marker, the drug developer proposes a numerical cut point. Then, patients with a high value of this biomarker (above the cut point) will be identified as “marker‐positive” patients. Otherwise, they are thought to be “marker‐negative” patients. For discussion of this manuscript, it is assumed that the “marker‐positive” subgroup benefits most from the test drug.

For enrichment strategy, the study design needs to make multiple comparison adjustments [6, 7]. A simple and commonly used adjustment is fixed‐sequence testing. The testing strategy can start with a demonstration of efficacy in the marker‐positive subgroup first; if successful, it then attempts to prove that the drug is also effective for the entire population. Alternatively, one could show drug efficacy for the entire population first, and if successful, then demonstrate that the efficacy is better in the marker‐positive subgroup. Other popular non‐parametric testing procedure includes Bonferroni, Hochberg [8] and Holm [9] procedure. Additionally, those popular multiplicity adjustment procedures can be presented or extended using the graphic approach [10, 11]. Furthermore, parametric multiple testing procedures can be used to exploit positive correlations among the population tests and further increase the power [12].

However, an important yet often unstated point is that the multiple comparison adjustment should not be the only consideration. It is also imperative to study the consistency between the marker‐positive subgroup and the marker‐negative subgroup. If the new drug is efficacious for the entire patient population, then it is expected to demonstrate consistent efficacy in both subgroups—it has to be beneficial to the marker‐positive patients, and beneficial to the marker‐negative patients. On the other hand, if the drug developer hopes to register the new drug only for the marker‐positive population, then there should be clear evidence that the test drug is efficacious in treating the marker‐positive patients, but the drug also should demonstrate that it does no harm to the marker‐negative patients. In addition, an estimation‐based assessment of the consistency based on the magnitude of treatment effect is preferred to support the approvability of this study drug [12]. Methods developed in this manuscript make use of this requirement of consistency to control for the multiple comparison adjustment. In most other multiple comparison adjustments, this requirement is either not considered in their approaches or not assessed based on the estimation of the magnitude of the treatment effect.

This paper is organized as follows. The mathematical details of the newly proposed method, which incorporates treatment consistency, are described in Section 2. Section 3 provides an example to illustrate the method, while Section 4 evaluates the power of the method through a simulation study. Finally, a thorough discussion of important considerations in enrichment design is provided in Section 5.

## 2 Methods

### 2.1 General Set Up

We assume patients can be either marker‐positive (S) or not (C, complement). Each patient either takes the placebo (0) or the treatment (1). This results in four groups, each with $n_{s,0},n_{s,1},n_{c,0},n_{c,1}$ patients, respectively. We assume the outcome of each patient is distributed according to

$$\begin{array}{ll} X_{s,0,i} & ∼N(\theta_{s,0},\sigma^{2})\\ X_{s,1,i} & ∼N(\theta_{s,1},\sigma^{2})\\ X_{c,0,i} & ∼N(\theta_{c,0},\sigma^{2})\\ X_{c,1,i} & ∼N(\theta_{c,1},\sigma^{2}) \end{array}$$

where i is a label for each patient in the corresponding group.

The sample mean outcome of each group, therefore, has a distribution

$$\begin{array}{ll} {\bar{X}}_{s,0} & ∼N(\theta_{s,0},\sigma^{2}/n_{s,0})\\ {\bar{X}}_{s,1} & ∼N(\theta_{s,1},\sigma^{2}/n_{s,1})\\ {\bar{X}}_{c,0} & ∼N(\theta_{c,0},\sigma^{2}/n_{c,0})\\ {\bar{X}}_{c,1} & ∼N(\theta_{c,1},\sigma^{2}/n_{c,1}) \end{array}$$

The sample variance is then

$${\hat{\sigma }}^{2}=\frac{1}{n_{s,0}+n_{s,1}+n_{c,0}+n_{c,1}-4}\underset{g\in \{(s,0),(s,1),(c,0),(c,1)\}}{\sum }\;\overset{n_{g}}{\underset{i=1}{\sum }}{\left(X_{g,i}-{\bar{X}}_{g}\right)}^{2}$$

One can show that the sample means and the pooled sample variance are independent, using ideas found in the proof of Theorem 5.3.1 of [13], with the pooled sample variance satisfying

$$(n_{all}-4){\hat{\sigma }}^{2}/\sigma^{2}∼\chi_{n_{all}-4}^{2}$$

where we define $n_{all}=\sum_{g}n_{g}$ to be the sum of sizes of all groups.

We now define

$$\theta_{s}=\theta_{s,1}-\theta_{s,0},\;\theta_{c}=\theta_{c,1}-\theta_{c,0},$$

and take

$${\hat{\theta }}_{s}={\bar{X}}_{s,1}-{\bar{X}}_{s,0},\;{\hat{\theta }}_{c}={\bar{X}}_{c,1}-{\bar{X}}_{c,0}$$

Then we have, independently,

(1) $$\begin{array}{ll} {\hat{\theta }}_{s} & ∼N\left(\theta_{s},\sigma^{2}\left(\frac{1}{n_{s,0}}+\frac{1}{n_{s,1}}\right)\right)\\ {\hat{\theta }}_{c} & ∼N\left(\theta_{c},\sigma^{2}\left(\frac{1}{n_{c,0}}+\frac{1}{n_{c,1}}\right)\right)\\ {\hat{\sigma }}^{2} & ∼\frac{\sigma^{2}}{n_{all}-4}\chi_{n_{all}-4}^{2} \end{array}$$

This can be used to generate samples of $\left({\hat{\theta }}_{s},{\hat{\theta }}_{c},{\hat{\sigma }}^{2}\right)$.

We specialize to the case $n_{s}=n_{s,0}=n_{s,1}$ and $n_{c}=n_{c,0}=n_{c,1}$. Then we have

$$\begin{array}{ll} {\hat{\theta }}_{s} & ∼N\left(\theta_{s},2\sigma^{2}/n_{s}\right)\\ {\hat{\theta }}_{c} & ∼N\left(\theta_{c},2\sigma^{2}/n_{c}\right)\\ {\hat{\sigma }}^{2} & ∼\frac{\sigma^{2}}{2n_{s}+2n_{c}-4}\chi_{2n_{s}+2n_{c}-4}^{2} \end{array}$$

### 2.2 Proposed Approach

We use normal endpoints to illustrate the new approach. Suppose a study has two primary hypotheses:

1. The treatment is better than the control in the overall population.
2. The treatment is better than the control in the marker‐positive subgroup S.

It is desirable for the drug to be approved if the treatment effect is significant in either the overall population or the marker‐positive population. Multiplicity adjustment is generally required under such situations for the family‐wise error rate to be under α. Recall that the estimated treatment differences for the marker positive subgroup, its complement, and overall population are denoted as ${\hat{\theta }}_{s}$, ${\hat{\theta }}_{c}$ and ${\hat{\theta }}_{o}$ respectively, with common subject level variance estimation for response as $\hat{\sigma }$. The test statistics are $T_{s}$, $T_{c}$ and $T_{o}$, with $n_{s}$ subjects per arm in marker‐positive subgroup, and $n_{c}$ subjects per arm in its complement, while the prevalence rate of the subgroup in the overall population is $p=n_{s}/(n_{s}+n_{c})$.

Therefore, the one‐sided hypothesis test may be written as

$$H_{0}^{s}:\theta_{s}\leq 0,\;H_{A}^{s}:\theta_{s}>0$$

$$H_{0}^{o}:\theta_{o}\leq 0,\;H_{A}^{o}:\theta_{o}>0$$

Motivated by the consistency‐based multiplicity adjustment proposed by Li et al. (2022) [14] and Alosh et al. (2009) [15], we define the decision rule for the marker‐positive subgroup hypothesis $H_{0}^{s}$ to restrict the performance of the treatment in the complementary subgroup. Statistical significance of the marker‐positive group can be claimed if

1. the p‐value from the test for the marker‐positive group is below $\alpha_{s}$, and
2. the treatment effect size for the marker‐negative group is more than a fraction b (−1<b<1) of the effect size for the marker‐negative group.

That means,

(2) $$\begin{array}{l} T_{s}>t_{\alpha_{s}}\;\text{and}\;\;{\hat{\theta }}_{c}>b\hat{\theta_{s}} \end{array}$$

where $t_{\alpha_{s}}$ is the $1-\alpha_{s}$ quantile of student's t distribution with degree of freedom $2n_{s}+2n_{c}-4$.

One natural choice is to use b=0. This means that in order for the marker‐positive subgroup to claim success, the drug should do no harm to the marker‐negative subgroup (numerically). Since there is always some level of classification error in defining subgroups, if a harmful effect is observed, even only for the marker‐negative subgroup, it may cause concern for the eventual approval of the treatment. When there is no true treatment effect ($\theta_{c}=0$), a negative point estimate may occur due to randomness, especially if the sample size for the marker‐negative subgroup is small. Then a b value between −0.3 and 0 may be considered.

On the other hand, the decision rule for the test of the overall population $H_{0}^{o}$ can be set up as

(3) $$\begin{array}{l} T_{o}>t_{\alpha_{o}}\;\text{and}\;\;{\hat{\theta }}_{s}>k{\hat{\theta }}_{o}\;\text{and}\;\;{\hat{\theta }}_{c}>k{\hat{\theta }}_{o} \end{array}$$

where $t_{\alpha_{o}}$ is the $1-\alpha_{o}$ quantile of student's t distribution with degree of freedom $2n_{s}+2n_{c}-4$. It indicates that the statistical significance of the overall population can be claimed if

1. the p‐value from the test for the overall population is below $\alpha_{o}$, and
2. the treatment effect sizes for both marker‐positive and marker‐negative groups are more than a fraction k (0<k<1) of the effect size in the overall population.

In this paper, the same k value is used for both subgroups since it is intended to describe the minimal treatment effect each subgroup needs to demonstrate so that it can be comfortably claimed that the treatment works in the overall population. If the two subgroups have different benefit‐risk evaluation profiles, and it is scientifically justified that the minimal threshold could be different for the two subgroups, however, then our method can be adapted to allow different k values to be used in the two subgroups.

Since $T_{o}$ is a deterministic function of $T_{s}$ and $T_{c}$, we write the decision rule in terms of $\left(T_{s},T_{c}\right)$. We take

(4) $$\begin{array}{l} T_{s}=\frac{\sqrt{n_{s}}{\hat{\theta }}_{s}}{\sqrt{2}\hat{\sigma }}∼t_{2n_{s}+2n_{c}-4},\;T_{c}=\frac{\sqrt{n_{c}}{\hat{\theta }}_{c}}{\sqrt{2}\hat{\sigma }}∼t_{2n_{s}+2n_{c}-4} \end{array}$$

Then we have

$$\sqrt{n_{s}/2}({\hat{\theta }}_{s}-\theta_{s})/\sigma ∼N(0,1)$$

$$\sqrt{n_{c}/2}({\hat{\theta }}_{c}-\theta_{c})/\sigma ∼N(0,1)$$

$$(2n_{s}+2n_{c}-4){\hat{\sigma }}^{2}/\sigma^{2}∼\chi_{2n_{s}+2n_{c}-4}^{2}$$

Therefore

$$\begin{array}{ll} T_{s} & =\frac{\sqrt{n_{s}/2}\theta_{s}+\sigma N(0,1)}{\hat{\sigma }}=\sqrt{2n_{s}+2n_{c}-4}\frac{N(0,1)+\sqrt{n_{s}/2}\frac{\theta_{s}}{\sigma }}{\sqrt{(2n_{s}+2n_{c}-4){\hat{\sigma }}^{2}/\sigma^{2}}}\\ T_{c} & =\sqrt{2n_{s}+2n_{c}-4}\frac{N(0,1)+\sqrt{n_{c}/2}\frac{\theta_{c}}{\sigma }}{\sqrt{(2n_{s}+2n_{c}-4){\hat{\sigma }}^{2}/\sigma^{2}}} \end{array}$$

We can rewrite the decision rule above in terms of only $T_{s}$ and $T_{c}$:

(5) $$\begin{array}{ll} \text{a.}\; & T_{s}>t_{\alpha_{s}},\;T_{c}>b\sqrt{(1-p)/p}T_{s}\\ \text{or b.}\; & \sqrt{p}T_{s}+\sqrt{1-p}T_{c}>t_{\alpha_{o}}\\ & kT_{c}<\left((1-k)\sqrt{p/(1-p)}+\sqrt{(1-p)/p}\right)T_{s}\\ & \;\left(\sqrt{p/(1-p)}+(1-k)\sqrt{(1-p)/p}\right)T_{c}>kT_{s} \end{array}$$

**FIGURE 1:**
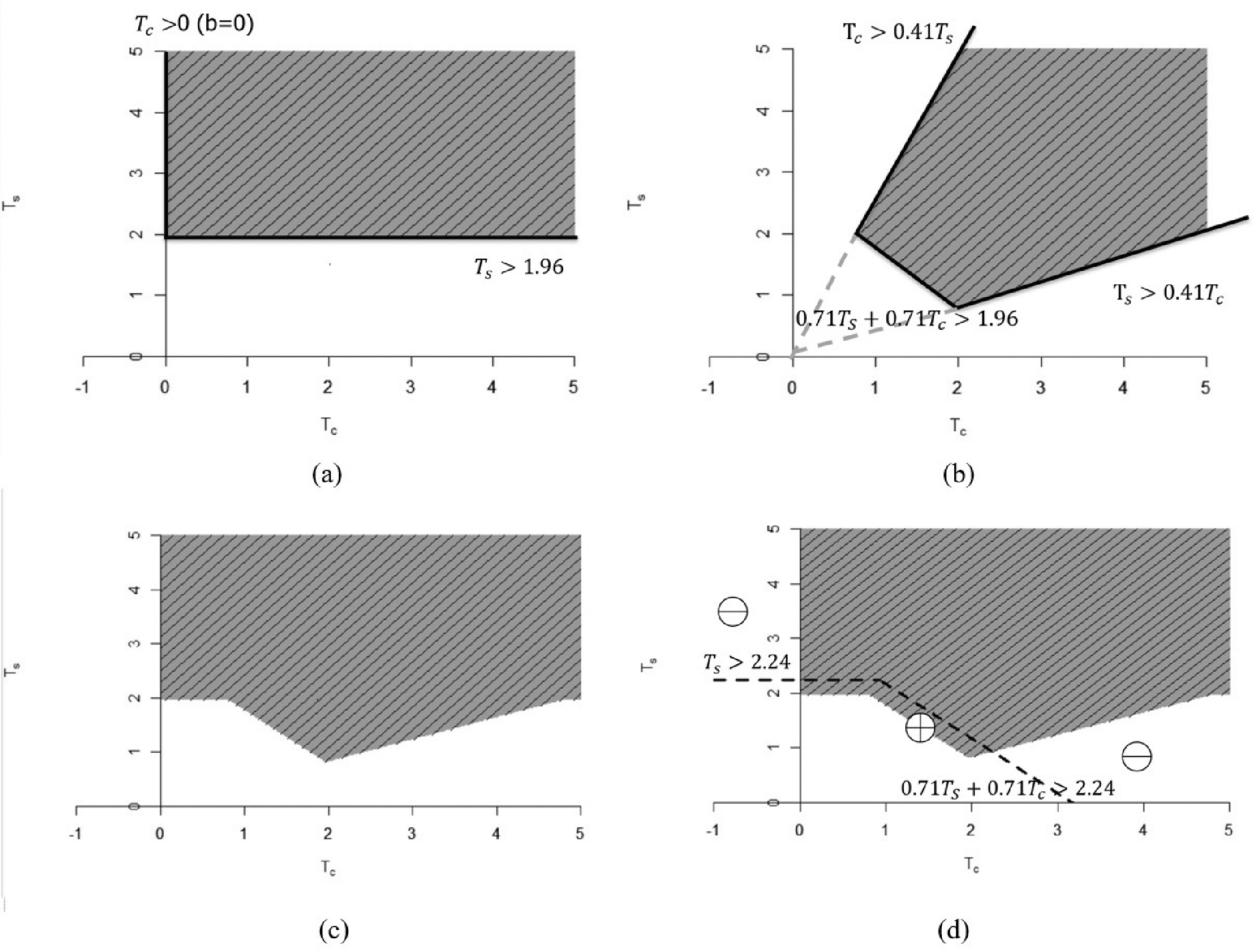
Rejection region for the proposed new method: (a) Rejection region to claim success for the marker‐positive group, (b) The rejection region to claim success for the overall population, (c) Rejection region to claim success of the study (success for the marker‐positive group or the overall population), and (d) Comparison of the new method with Bonferroni Adjustment.

**FIGURE 2:**
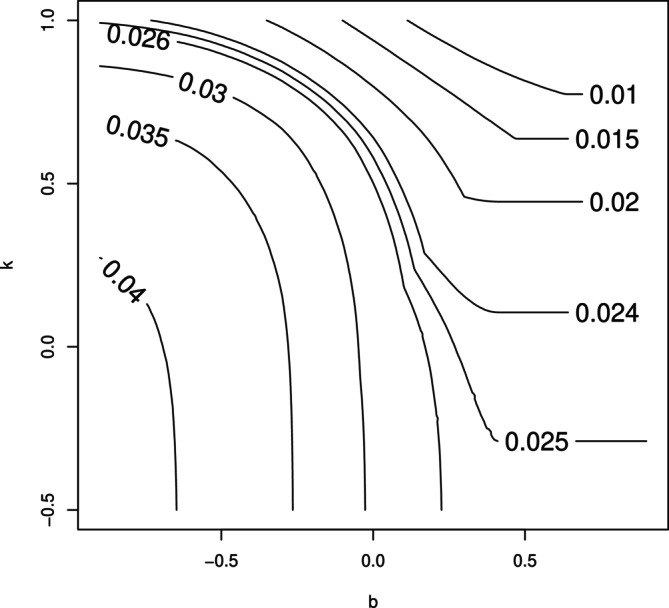
The combination of b values and k values for different α when $\alpha_{s}=\alpha_{c}=0.025,p=0.5$.

For given values of $\alpha_{s},\alpha_{o},p,b$ and k, type I error for testing the intersection hypothesis of $H_{0}^{s}\cap H_{0}^{o}$ can be computed by evaluating the probability of the rejection region as stipulated by (5). As shown in Figure 1, the rejection region can take a somewhat complicated shape. In this case, Monte‐Carlo integration is easier to implement. To this end, we first generate a larger number (say $10^{6}$) of samples of $\left({\hat{\theta }}_{s},{\hat{\theta }}_{c},{\hat{\sigma }}^{2}\right)$ according to (1), which is then used to generated samples of $\left(T_{s},T_{c}\right)$ according to (4). We use these samples to compute the probability of rejection. Conversely, given values of $\alpha_{s},\alpha_{o},p$ and b, the value of k that achieves the desired type I error can also be computed. The combination of b values and k values and the corresponding type I error rate α (contour value) for the intersection hypothesis is given in Figure 2 for $\alpha_{s}=\alpha_{o}=0.025,p=0.5$. More combinations are provided in Table 1. This approach has taken into account the correlation between $T_{s}$ and $T_{c}$ to reduce the multiplicity penalty, similar to the Dunnett test.

**TABLE 1:** The combination of b values and k values at type I error rate α=0.025 for given $\alpha_{s}$, $\alpha_{c}$, and p.

Proportion of marker‐positive p	Alpha level^a^		Required % of effect size retained^b^	
	$\alpha_{s}$	$\alpha_{o}$	b	k
0.5	0.025	0.025	−90%	99%
0.5	0.025	0.025	−50%	93%
0.5	0.025	0.025	0	59%
0.5	0.025	0.025	8%	43%
0.5	0.025	0.025	20%	15%
0.5	0.025	0.025	50%	−15%

^a^ *Note*: Alpha level for marker‐positive and the overall population, respectively.

^b^ Required proportion of effect size from the marker‐positive group to be retained in the marker‐negative group (b) and required proportion of effect size from the overall population to be retained in both marker‐positive and marker‐negative groups (k).

It is worth noting that the decision rules in Condition (5) are for testing the intersection hypothesis of $H_{0}^{s}\cap H_{0}^{o}$. Based on closed testing principle [16], if the intersection hypothesis is rejected, each individual hypothesis can be tested at the full α level, and this process maintains strong control over the family‐wise error rate. The way the decision rule is constructed also implies that once the Condition (5) for rejecting the intersection hypothesis is satisfied, at least one hypothesis from $H_{0}^{s}$ and $H_{0}^{o}$ will be rejected. Therefore, Condition (5) also defines the success of the study. Another important implication is that if only one condition (5) a or b is satisfied in Condition (5), it does not mean that the other population that fails to pass can not claim success. For example, if $T_{o}$ is 0.023 with $\alpha_{o}=0.0125$ in Condition (5), the overall population will not pass the intersection test criterion in Condition (5b). However, as long as Condition (5a) is satisfied, the intersection hypothesis will be rejected. $T_{o}$ can then be tested at α=0.025 level and it will pass the test. In this case, the overall population can still claim statistical significance even if Condition (5b) is not satisfied.

**FIGURE 3:**
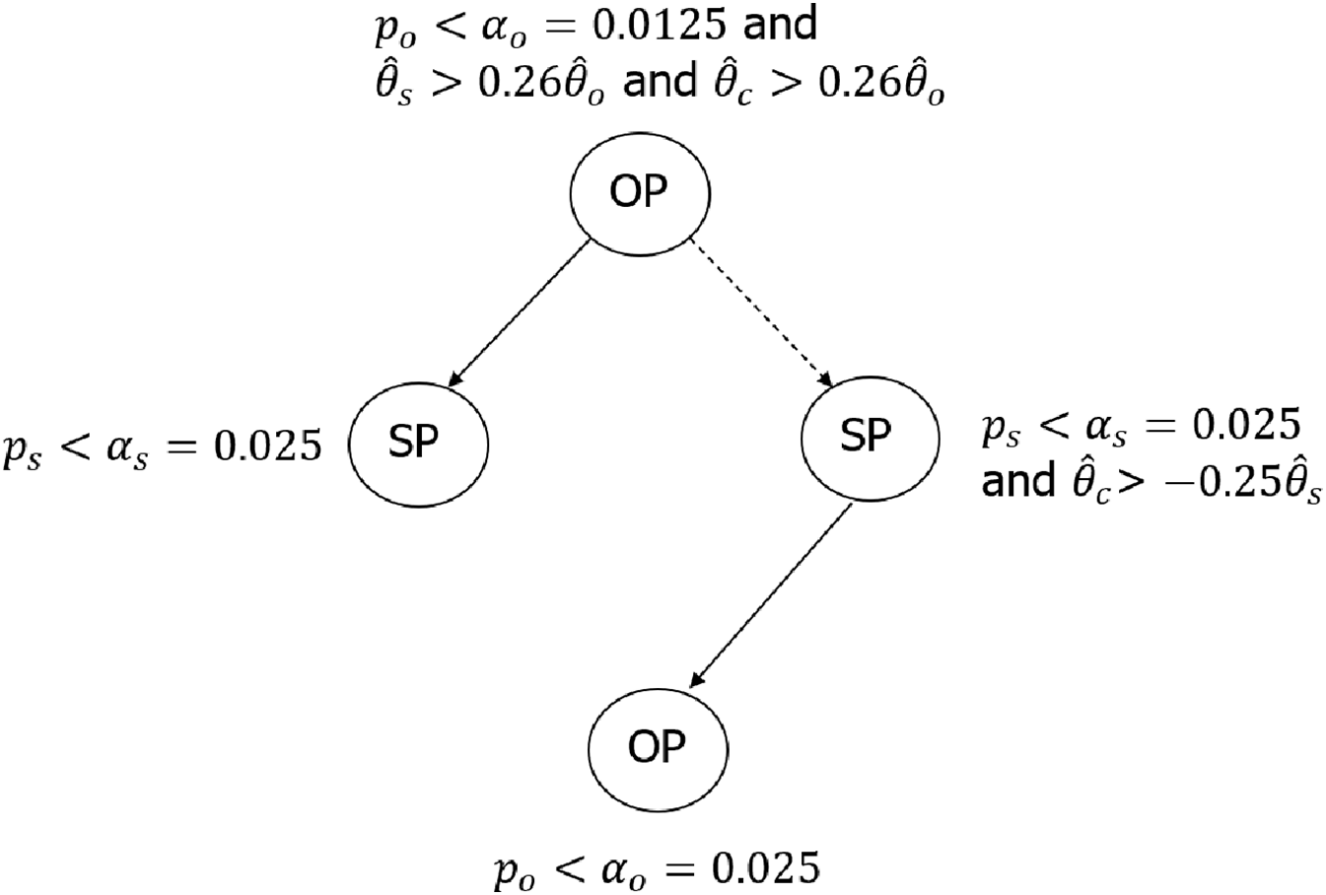
Testing strategy with two patient populations, illustrated using step‐wise diagram. Patient populations: OP, overall population; SP, subpopulation/marker‐positive population. Decision paths: —, significant outcome; – – –, non‐significant outcome.

**FIGURE 4:**
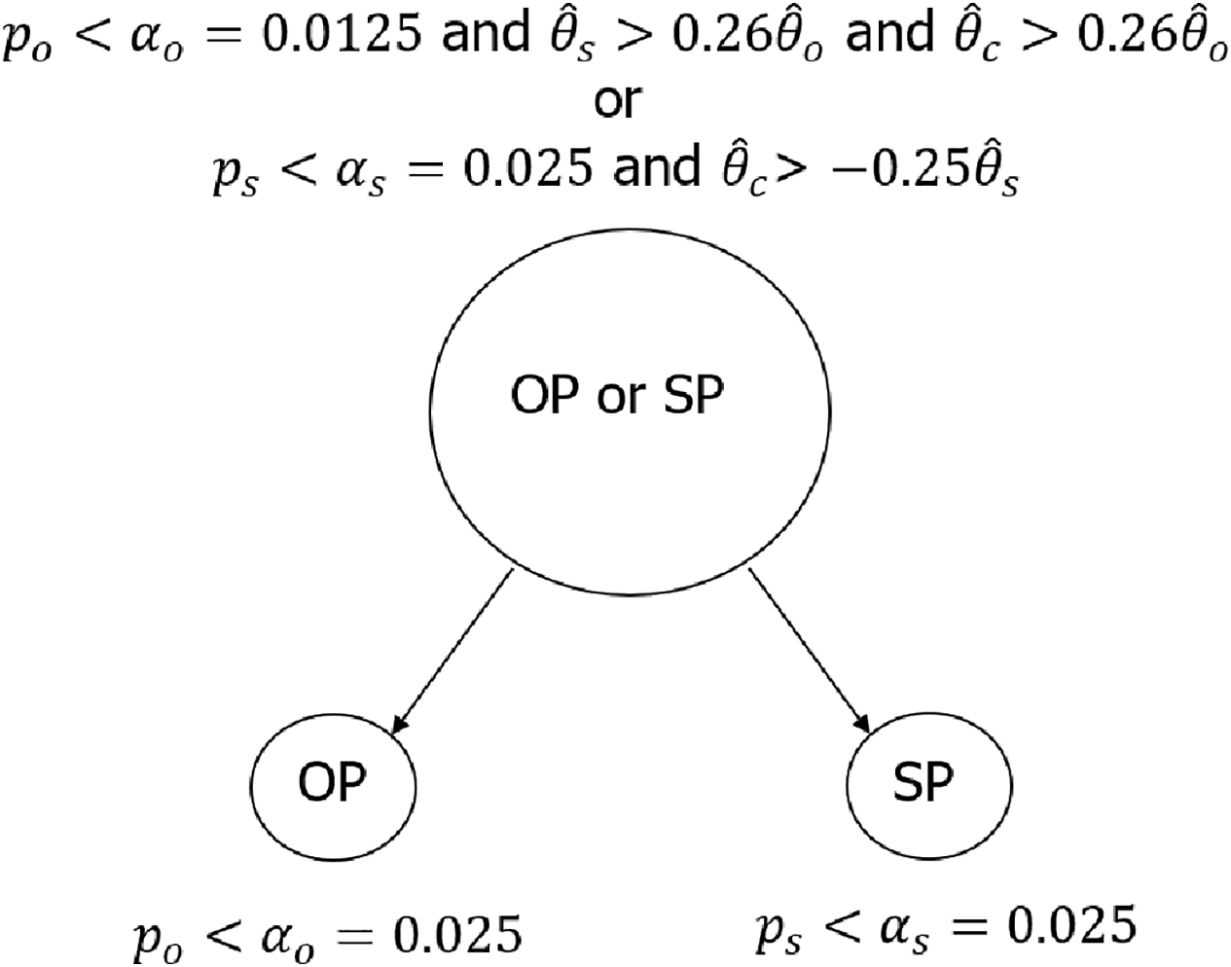
Testing strategy with two patient populations, illustrated using close‐testing procedure. Patient populations: OP, overall population; SP, subpopulation/marker‐positive population. Decision paths: —, significant outcome; – – –, non‐significant outcome.

As an example, the multiplicity testing procedure using b=−0.25 and k=0.26 can be illustrated using the graphic approach [11] in several ways. Figure 3 illustrates the testing strategy from this design using a step‐wise diagram. Figure 4 illustrates the same testing strategy using a closed‐testing procedure. It is clearer from Figure 4 that the tests for the overall population and marker‐positive population are essentially going parallel, while Figure 3 may be easier to understand for non‐statisticians. In Figure 4, although not required from a type I error control perspective, similar consistency criteria in condition (5)—such as $T_{c}>b\sqrt{(1-p)/p}T_{s}$ for marker positive population (SP)—may be applied into the second layer of testing. This version of the method will be referred to as the “constrained” approach in Section 4, where simulation results are discussed.

## 3 Rejection Region Illustrated by an Example

Now, the rejection region of the new method will be illustrated by an example. Suppose we have a study where the marker‐positive subgroup represents 50% of the overall study population (p=0.5). Let $n_{s}=n_{c}=150,\alpha_{s}=\alpha_{o}=0.025,t_{\alpha_{s}}=t_{\alpha_{o}}=1.96$, which means the tests for overall population and marker‐positive subgroup are both done at full alpha level. Let b=0, which means that we require the marker‐negative group not to harm to claim success for the marker‐positive group. Then k=0.59, which means this method requires both marker‐negative and marker‐positive groups to achieve at least 59% of the effect observed in the overall population to claim success for the overall population. Then the decision rule for the success of the study can be simplified as

$$T_{s}>1.96\;\text{and}\;{\hat{\theta }}_{c}\geq 0\;\text{equivalently as}\;T_{s}>1.96\;\text{and}\;T_{c}\geq 0$$

Or

$$T_{o}>1.96\;\text{and}\;{\hat{\theta }}_{s}>0.59{\hat{\theta }}_{o}\;\text{and}\;\;{\hat{\theta }}_{c}>0.59{\hat{\theta }}_{o}$$

Based on Equation (5b) this is equivalent to

$$0.71T_{s}+0.71T_{c}>1.96\;\;\text{and}\;\;T_{c}>0.41T_{s}\;\;\text{and}\;\;T_{s}>0.41T_{c}$$

**FIGURE 5:**
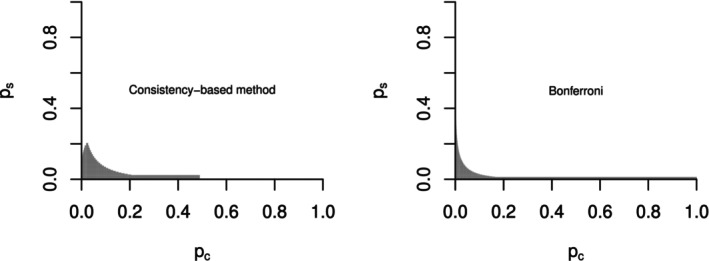
Rejection region based on p‐value scale. **Left**: The proposed consistency‐based method. **Right**: Bonferroni Adjustment.

The rejection region is shown in Figure 1. In Figure 1d, the rejection region based on Bonferroni correction is the area above the dashed line. The success of the study requires either $T_{s}>1.96$ or $T_{o}>1.96$, where the latter one is equivalent to $0.71T_{s}+0.71T_{c}>2.24$. Compared with the Bonferroni adjustment, the new method prioritizes the region where the marker‐positive and marker‐negative subgroups demonstrated consistent treatment effects. This is marked by the shaded area marked by the + sign in the upper right quadrant below the dashed line. As a trade‐off, the white area marked by the − sign between the dashed line and the shaded area is surrendered. The idea is that if the treatment seems to be harmful in the marker‐negative subgroup, the treatment may not be approved. Similarly, if the marker‐positive subgroup didn't sustain a reasonable portion of the effect observed in the overall population, then the claim in the overall population can't be made. This is consistent with the actual decision‐making process in regulatory approval that is beyond the consideration of p‐value. Although the trade‐off may be perceived as surrendering a larger area for a small area in the plot based on test statistic T values, it is worth noting that the probability density is not homogeneous in Figure 1. Another way to look at this is to display the rejection region on the p‐value scale. Since the p‐value is uniformly distributed between 0 and 1 under the null hypothesis, the areas will be directly comparable (Figure 5).

## 4 Simulation and Power

Suppose there is a test drug that is expected to yield a clinically meaningful treatment effect size ($\theta_{s}$) of 0.3 on a normally distributed endpoint in the marker‐positive subgroup. Without loss of generality, let's assume σ=1. With 150 patients in the placebo and test drug, respectively, a two‐arm parallel design study with type I error rate α=0.025 (one‐sided) will yield a power of 73.6%. With 300 patients in the placebo and test drug, respectively, the power will be 95.6%. Now, suppose the phase II study focused on the marker‐positive population and demonstrated a standardized effect size of 0.3. The phase II study also enrolled a small marker‐negative subgroup, in which a reduced effect size of 0.2 was observed. Due to the small sample size in the phase II study, it is unclear if the drug will perform similarly well in the marker‐negative subgroup. The prevalence rate is only 30% for the marker‐positive patients in the overall population. To mitigate the risk, an enrichment design is proposed to make sure at least 50% of the subjects enrolled are marker‐positive in the phase III study. Meanwhile, some marker‐negative patients are included to provide important information regarding the effectiveness in the overall population. Next, a few design options are explored and compared.

**FIGURE 6:**
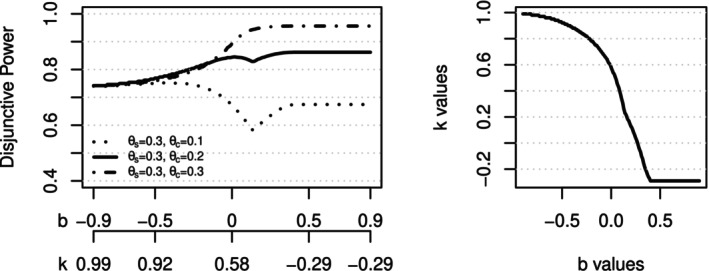
Design with $n_{s}=n_{s,0}=n_{s,1}=150$ in marker‐positive subgroup and $n_{c}=n_{c,0}=n_{c,1}=150$ in marker‐negative subgroups (p=0.5). $\alpha_{s}=\alpha_{o}=0.025$. **Left**: Disjunctive power with either overall population or marker‐positive subgroup claiming statistical significance. **Right**: b‐value and k‐value combinations to yield a type I error of 0.025 for the intersection test.

If the phase III study is designed with $n_{s}=n_{s,0}=n_{s,1}=150$ in marker‐positive subgroup and $n_{c}=n_{c,0}=n_{c,1}=150$ in marker‐negative subgroups, with $\alpha_{s}=\alpha_{o}=0.025$ and p=0.5, then the disjunctive power with either overall population or marker‐positive subgroup passing intersection test is displayed in Figure 6 on the left. With increasing b values, the k values will decrease so that the overall type I error for the test of the intersection hypothesis for the overall population and marker‐positive subgroup is maintained at α=0.025. The k values for each value of b are displayed on the right side of Figure 6). When b‐values approach −1, there is essentially little restriction on the effect size of the marker‐negative subgroup to pass the intersection test for the marker‐positive subgroup. As a trade‐off, the k values approach 1, which means that to pass the intersection test for the overall population, both marker‐positive and marker‐negative subgroups need to demonstrate almost identical effect sizes as in the overall population. That essentially diminishes the possibility of passing the effect size threshold in Equation (5b). It means that the consistency‐based method resembles the fixed‐sequence testing procedure to test the marker‐positive subgroup first, and only move on to test for the overall population if the first test is passed. As discussed before, the power for this testing strategy should be around 73.6%. Similarly, when b values increase to be around 0.4, it will be very difficult to pass the intersection test for the marker‐positive subgroup. The k values approach −0.3, and then stay there. This setup resembles the fixed‐sequence testing procedure to test the overall population first, and only move on to test for the marker‐positive subgroup if the first test is passed. The power for this testing strategy should be around 95.6%.

**FIGURE 7:**
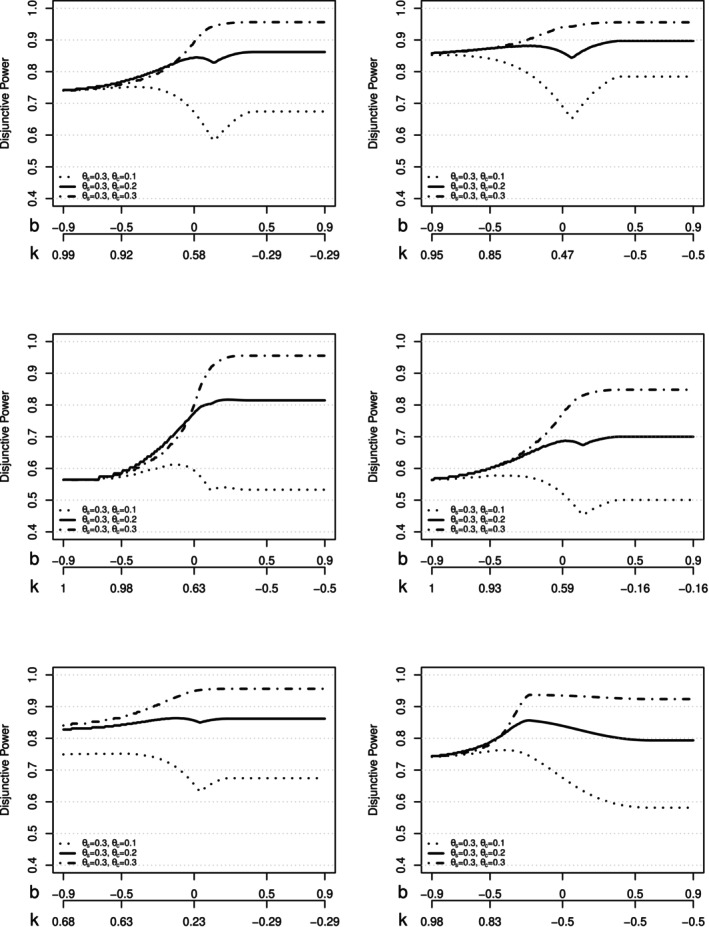
Disjunctive power for six scenarios: **Top left**: Design 1 with $n_{s}=150,n_{c}=150$, $\alpha_{s}=\alpha_{o}=0.025$. **Top right**: Design 2 with $n_{s}=200,n_{c}=100$, $\alpha_{s}=\alpha_{o}=0.025$. **Middle left**: Design 3 with $n_{s}=100,n_{c}=200$, $\alpha_{s}=\alpha_{o}=0.025$. **Middle right** Design 4 with $n_{s}=100,n_{c}=100$, $\alpha_{s}=\alpha_{o}=0.025$. **Bottom left**: Design 5 with $n_{s}=150,n_{c}=150$, $\alpha_{s}=0.0125,\alpha_{o}=0.025$. **Bottom right**: Design 6 with $n_{s}=150,n_{c}=150$, $\alpha_{s}=0.025,\alpha_{o}=0.0125$.

Regarding the power of the proposed method, b values between 0.1 to 0.4 should be avoided since it leads to the lowest power when the effect size in the marker‐negative subgroup is small to moderate, while it doesn't increase the power when the marker‐negative subgroup is similar to the marker‐positive subgroup. Meanwhile, b values between −0.3 to 0.1 lead to a different trade‐off between marker‐positive and overall population. The bigger the b values, the smaller the k values, and the more chances of success are shifted to the overall population compared to the marker‐positive population. Not surprisingly, it also makes the method more sensitive to the variation of the marker‐negative subgroup's effect size. Therefore, if there is data to support a strong prior belief about the efficacy of the test drug on the marker‐negative subgroup, a larger b around 0 to 0.1 may be chosen to boost the power of the overall population. Otherwise, a smaller and slightly negative b value should be chosen to maintain the robustness of the design. A similar pattern is observed in other scenarios, as in Figure 7.

A few other scenarios are explored for disjunctive power and presented in Figure 7. The top left is Design 1 with the same setting as Figure 6 for reference. Top right is Design 2 with $n_{s}=200$ in marker‐positive subgroup and $n_{c}=100$ in marker‐negative subgroups, so two‐thirds of the study population is marker‐positive (p=2/3) while the overall sample size is the same with Design 1. $\alpha_{s}=\alpha_{o}=0.025$. Since the proportion of the marker‐negative group is lower in this setting, the power is not as sensitive to the variations of the marker‐negative group's effect size. When the effect size for the marker‐negative group is small, the small sample size in the group reduces the damage. When the effect size for the marker‐negative group is high, the overall power is already high, and the marginal increase in power is small. In contrast, Design 3 with $n_{s}=100$ in the marker‐positive subgroup and $n_{c}=200$ in the marker‐negative subgroups is displayed on the middle left. Only one‐third of the study population is marker‐positive, and $\alpha_{s}=\alpha_{o}=0.025$. In this case, disjunctive power is very sensitive to the marker‐negative group's effect size, and the power is not going to be sufficient unless the marker‐negative subgroup has a similar effect as the marker‐positive. Such a design should be avoided if there is no strong data supporting the efficacy of the marker‐negative subgroup. The middle right of Figure 7 shows the Design 4 with $n_{s}=100$ in marker‐positive and marker‐negative subgroup respectively and $\alpha_{s}=\alpha_{o}=0.025$. It demonstrated that if the sample size is only calculated based on the assumption that the marker‐negative works equally well as the marker‐negative subgroup, and the overall population is used for the primary test, it is likely that the study will be underpowered if the assumption is deviated.

Finally, the bottom left is Design 5 with $n_{s}=150$ in the marker‐positive subgroup and $n_{c}=150$ in the marker‐negative subgroup. Unbalanced α is used for the two tests with $\alpha_{s}=0.0125,\alpha_{o}=0.025$. The design noticeably reduces the additional effect size threshold (b and k) in Equations (2) and (3). For example, for b=0, the k value is decreased from 0.58 to 0.23. It also increases the power, especially when the marker‐negative effective size is moderate or comparable to the marker‐positive subgroup. On the bottom right is the Design 6 where $\alpha_{s}=0.025,\alpha_{o}=0.0125$. Design 6 reaches good power around b=−0.25 and k=0.26 for all three setups with small, moderate, and target effect sizes for the marker‐negative group. This design leads to 93% power when $\theta_{c}=0.3$, 86% power when $\theta_{c}=0.2$ and 75% power when $\theta_{c}=0.1$.

Table 2 presents the comparison of the proposed method under different setups when b=−0.25 and the total sample size is $n_{s}+s_{c}=300$. Design 6 almost reaches the highest disjunctive power for all three marker‐negative effect sizes across design options, and is therefore preferred. When the effect size for the marker‐negative group is small $\theta_{c}=0.1$, the disjunctive power for Design 6 (75%) is slightly higher than the fixed‐sequence test starting with the marker‐positive subgroup. When the effect size for the marker‐negative group reaches target $\theta_{c}=0.3$, the disjunctive power for Design 6 (93%) approaches the method with a fixed‐sequence test starting with the overall population. The only more robust design is Design 2, which requires an increased proportion of marker‐positive participants, which may not be feasible or delay the development timeline if the prevalence of the marker‐positive group is low in the natural population.

**TABLE 2:** Comparison of disjunctive power for different designs when b=−0.25 and total sample size is 300.

	Design parameter						Disjunctive power		
	$n_{s}$	$n_{c}$	$\alpha_{s}$	$\alpha_{o}$	b	k	$\theta_{c}=0.1$	$\theta_{c}=0.2$	$\theta_{c}=0.3$
Design 1	150	150	0.025	0.025	−0.25	0.82	0.74	0.80	0.79
Design 2	200	100	0.025	0.025	−0.25	0.73	0.80	0.88	0.90
Design 3	100	200	0.025	0.025	−0.25	0.89	0.60	0.66	0.64
Design 5	150	150	0.0125	0.025	−0.25	0.54	0.74	0.86	0.91
(Preferred) Design 6	150	150	0.025	0.0125	−0.25	0.26	0.75	0.86	0.93

In addition, Design 6 is further compared with Bonferroni and Dunnett multiplicity adjustment in Table 3. Although the Dunnett test [17] is more powerful than Bonferroni by leveraging the correlation between the test statistics of the overall population and marker‐positive subgroup, its disjunctive power is just comparable to Design 6. As discussed in previous sections, it is also important to note that although the nominal disjunctive power may appear similar, the Bonferroni and Dunnett tests may gain part of their power from the rejection region associated with a negative effect in the marker‐negative population. This extreme scenario could raise regulatory concerns about data consistency and potentially hinder approval, even if statistical significance is achieved in the marker‐positive or overall population. Another, more likely scenario is one in which the marker‐negative population exhibits a minimal treatment effect that is not clinically meaningful, with positive outcomes in the overall population driven primarily by a strong effect in the biomarker‐positive subgroup. Such disparities could similarly raise concerns during the approval process, even if statistical significance is achieved in the overall population. If we exclude those scenarios based on consistency criteria similar to Design 6, the resulting power decreases to the levels observed under the “Constrained Bonferroni” and “Constrained Dunnett”. These constrained approaches show up to a 5% loss in power compared to the proposed method. This result is not surprising, as the proposed method not only leverages the correlation structure similar to the Dunnett approach, but also incorporates the consistency requirement directly into the construction of the rejection region.

**TABLE 3:** Comparison of power for Design 6 with Bonferroni and Dunnett method with sample size 150 per arm.

	Power (marker positive)			Power (overall)			Disjunctive power		
	$\theta_{c}=0.1$	$\theta_{c}=0.2$	$\theta_{c}=0.3$	$\theta_{c}=0.1$	$\theta_{c}=0.2$	$\theta_{c}=0.3$	$\theta_{c}=0.1$	$\theta_{c}=0.2$	$\theta_{c}=0.3$
Design 6	0.70	0.73	0.74	0.65	0.83	0.93	0.75	0.86	0.93
Constrained design 6	0.70	0.73	0.74	0.58	0.81	0.93	0.75	0.86	0.93
Bonferroni	0.64	0.64	0.64	0.58	0.79	0.92	0.73	0.84	0.93
Constrained Bonferroni	0.61	0.63	0.64	0.53	0.78	0.92	0.70	0.83	0.93
Dunnett	0.66	0.66	0.66	0.61	0.81	0.93	0.75	0.85	0.94
Constrained Dunnett	0.63	0.66	0.66	0.55	0.80	0.93	0.72	0.85	0.94

In addition, the proposed approach yields better power for each individual test—both for the marker‐positive population and the overall population. Specifically, Design 6 demonstrates considerably higher power for the marker‐positive subgroup due to the increased $\alpha_{s}=0.025$, even when compared to the unconstrained Bonferroni and Dunnett approaches. The power for the overall population is also slightly higher under the proposed approach, particularly when the treatment effect is heterogeneous. This improvement is likely attributable to the ability to re‐test the overall population at the full alpha level when the intersection test is successful, as discussed in the final two paragraphs of Section 2.

## 5 Discussion

The consistency‐based approach strategically places the rejection region in an area where consistency is demonstrated, as opposed to an area lacking it. This method effectively harnesses the frequently neglected properties of clinical trials in the enrichment study design, which are often overlooked by standard multiplicity comparison procedures (MCPs). The advantage of not having to divide alpha between the overall population and the marker‐positive population comes with the trade‐off of imposing additional restrictions on the decision rules. However, the additional restrictions imposed on both tests‐for the marker‐positive subgroup and for the overall population‐mirror the anticipated treatment effect in the two populations. If these expectations hold true, this approach may yield greater power compared to the Bonferroni method. When tests are multi‐dimensional, it is not possible to have a uniformly most powerful MCP for all alternatives. The choice of an appropriate MCP at the trial design stage should be based on the probability of technical success by identifying a set of likely alternatives. The distributions of the likely alternatives can be estimated based on results from early‐phase studies.

The application of the consistency‐based approach enables statisticians to think more clearly. The power of the study depends not only on the effect size in the marker‐positive and marker‐negative subgroups, but also on several other factors. Considerations include the enrichment ratio of the marker‐positive subjects in the overall sample size and the restriction factors imposed on the decision rules. Plots and tabulations of various designs, along with the expected alternatives and corresponding power, can help identify a design that is robust to a range of likely alternatives.

In an enrichment clinical trial, recruiting marker‐negative patients could potentially “dilute” the treatment efficacy of the overall patient population. However, if the drug is, in fact, efficacious to both marker‐positive and marker‐negative patients, then the overall patient population can benefit from this study drug too. Strategically speaking, it is to the advantage of the drug developer to recruit both marker‐positive and marker‐negative patients into this enrichment clinical trial. With such a design, recruiting mostly marker‐positive patients, while also including some marker‐negative patients could maintain high efficacy among the overall participating patients. In sample size calculation, with a smaller portion of marker‐negative patients, the required sample size of the entire study could be less than that including a larger portion of marker‐negative patients. This is because in the first case, a larger expected treatment difference (against the control group) can be applied in the sample size calculation.

In addition to the choice of the sample size and the enrichment ratio of the biomarker‐positive subgroup in the overall population, the choices of the restriction factors (b and k) should also take into consideration the performance of the diagnostic companion instrument. It is also important to consider the population's prevalence of the biomarker‐positive subgroup. Even when the companion diagnostic instrument exhibits up to 80% sensitivity and specificity, the marker‐positive subgroup may still include a significant number of false‐positive patients due to the low positive predictive value. In these instances, the selection of the b value might need to account for the potential proportion of marker‐negative subjects who are misclassified as biomarker‐positive. Given the substantial impact these marker‐negative subjects can have on the power of the study, it could be beneficial to invest more resources in understanding the treatment effect within this group during early‐phase studies.

This consistency‐based approach opens many possible applications and potential alterations. For example, it is possible to use absolute values instead of a percentage of relative effect size, as the restrictions in the decision rules of the tests for the overall population and subgroup(s). Although such an alteration may need further evaluation, the interpretation and clinical meaning may be easier to understand than using the fractions in certain circumstances. In addition, the application may extend to endpoints beyond continuous and normally distributed endpoints. Similar restrictions can be imposed in the decision rule for the categorical and survival endpoints. Simulation can be used to determine the restrictions as well as to understand the relationship between the restrictions.

## Conflicts of Interest

The authors declare no conflicts of interest.

## Data Availability

The data that support the findings of this study are available from the corresponding author upon reasonable request.
